# Psychological Symptoms in Italian, Spanish and Portuguese Youth During the COVID-19 Health Crisis: A Longitudinal Study

**DOI:** 10.1007/s10578-021-01211-9

**Published:** 2021-06-26

**Authors:** Mireia Orgilés, Rita Francisco, Elisa Delvecchio, José P. Espada, Claudia Mazzeschi, Marta Pedro, Alexandra Morales

**Affiliations:** 1 Department of Health Psychology, Universidad Miguel Hernández, Avda. de La Universidad S/N, Elche, 03202 Alicante, Spain; 2 Università Degli Studi Di Perugia, Perugia, Italy; 3 Universidade Católica Portuguesa, Porto, Portugal

**Keywords:** Youth, Stress, COVID-19, Psychological impact, Longitudinal

## Abstract

Aiming to slow down the spread of the COVID-19, a lockdown was declared in the first term of 2020 in many European countries, applying different restrictions measures. Although the psychological effects of home confinement in children have been described, there is a lack of longitudinal research examining the impact of the confinement over time. The present study analyzes the evolution of the psychological wellbeing of children and adolescents from three European countries with different restrictions. Parents of 624 Italian, Spanish, and Portuguese children and adolescents aged 3 to 18 years old completed the “Impact Scale of COVID-19 and Home Confinement on Children and Adolescents” two, five, and eight weeks after the lockdown. Results show a different pattern for each country. Children from Italy, the first European country that applied a lockdown, were better adapted than Spanish and Portuguese children the first two weeks after confinement but they were more psychologically impacted by home confinement at the eight-week assessment. Portuguese children, who followed a general duty of home confinement, were the best adapted to the situation, with no significant differences over time. A significant change was found in anxiety symptoms in Spanish children, with a decrease at the last assessment. Findings suggest that long confinements and hard restrictions affect children, so prevention measures should be applied during confinements to prevent psychological problems in children.

## Introduction

On March 11th, 2020, the World Health Organization (WHO) declared the coronavirus disease (COVID-19) as a pandemic. The disease emerged in the city of Wuhan (China) in December 2019, but it spread rapidly, first in Italy, and then in other European countries. With more than 479,000 and 272,000 cases, respectively, Spain and Italy are among the five most affected countries in Europe as of September 3rd (European Centre for Diseases Prevention and Control, 2020). Portugal, however, despite being close to Spain, was only affected by 58,600 cases on the same date and was one of the countries worldwide that best managed to face the COVID-19 situation. Aiming to slow down the spread of the infections, a lockdown was declared in these three European countries, but different restrictions were applied. Italy declared the lockdown on March 10th in the entire country, restricting traveling only to work and attending emergencies and closing all the businesses, except for those providing essential services. On March 31st, after 21 days of home confinement, Italian children were allowed to take walks close to home with one adult for the first time, and on May 4th, parks were reopened and visits to relatives were allowed. Most businesses in Italy opened on May 18th, swimming pools and gyms on May 25th, and theatres and cinemas on June 15th. Spain imposed a lockdown six days after Italy, on March 16th, mandating all citizens to remain at home except for essential needs, such as purchasing food or medicines or attending health emergencies. On April 26th, after seven weeks of mandatory home confinement, Spanish children were allowed to go outside to take a walk no more than 1 km from home, one hour a day, once a day, and accompanied by only one adult. Restrictions were less harsh in Spain as of May 11th, starting a de-escalation process that finished on June 21st, when the State of Alarm expired and the country entered a “new normality” phase. Contrary to Italy and Spain, where home confinement was mandatory, in Portugal, confinement was only mandatory for patients with COVID-19 and those infected with SARS-Cov2, as well as for citizens under active surveillance. The schools closed on March 13th, and on March 18th, the Portuguese government declared the country to be in the State of Emergency. This implied restrictions on the right of movement, including the general obligation of home confinement. However, they were allowed, for example, to travel to public spaces and on roads to acquire goods and services, to perform professional or similar activities (including if they were looking for a job), for health reasons, to assist vulnerable people, to accompany minors on short trips to “enjoy the outdoors”, short trips for physical activity (although collective physical activity was prohibited) or to exercise pets. On May 2nd, the Emergency Status was canceled, and restrictions started to be removed progressively.

Although different rules were applied, home confinement affected the lives of Italian, Spanish, and Portuguese families. Parents had to deal with many stressors during the lockdown, such as taking care of their children´s learning, working from home, or managing the household [1–3]. Children also had to face the stress derived from the pandemic [4–6]. School closure changed children’s academic routines, and the social distance limited their relationships to indoors. The psychological effects of home confinement in European children have been described, finding emotional and behavioral problems during the COVID-19 confinement. Orgilés et al. [7] found that Italian and Spanish children changed their emotional and behavioral state during the confinement, showing boredom, difficulty concentrating, irritability, restlessness, nervousness, feelings of loneliness, uneasiness, and worries as the most frequent symptoms. Francisco et al. [8], in a sample of Spanish, Italian, and Portuguese children, reported that having an outdoor space (e.g., garden, terrace) was related to better psychological and behavioral wellbeing. Pisano et al. [9], in an Italian sample of children from 4 to 10 years old, concluded that the COVID-19 situation had an important effect on children’s wellbeing during the first month of confinement, showing a higher demand for physical proximity with their parents at night, irritability, and mood changes, among other problems. Studies with non-European samples also found that COVID-19 confinement impacted children negatively, as shown in a sample of Chinese children, which reported that 19% and 23%, respectively, had anxiety and depression symptoms after 34 days of confinement [10].

Despite that previous studies have examined the effects of home confinement on children’s mental health, to date, there is a lack of research on how the psychological effects of confinement due to COVID-19 have changed across time. The present study aims to analyze the evolution of the immediate psychological effects of the COVID-19 health crisis in children from countries with different levels of restrictions (Italy, Portugal, and Spain) using three time-points (two, five, and eight weeks after starting the lockdown). Specifically, the objectives of the study are: (a) to examine the variations in children’s psychological well-being (anxiety, mood, sleep, behavioral, eating, and cognitive areas) two, five, and eight weeks after the lockdown; (b) to analyze the evolution of the psychological effects in children from each country over time in the three time-points; and c) to study the interaction between each country and measurement time in the children’s psychological symptoms. In line with previous studies [7, 11, 12], it is hypothesized that the psychological effects on children will be higher the longer the home confinement and in those countries with more restrictions applied.

## Methods

### Participants

Parents of 624 children and adolescents aged 3–18 years old from Italy, Spain, and Portugal participated in the study. The profile of the sample is described in Table 1. Most parents were females and married, with a mean age of 42 years (*SD* = 5.48). Their children’s mean age was 8.94 years (*SD* = 4.19), and 52.9% were boys. No differences were observed in the sociodemographic variables between respondents from the three countries, except for the caregiver’s gender, educational level, and the children’s age. Compared to Spain, a higher proportion of females than males completed the online survey in Italy. The Portuguese sample included a higher proportion of participants holding a Ph.D. or Master’s degree, compared to the Italian one. A higher proportion of Italian participants had secondary studies, compared to the Portuguese. In Spain, there was a higher proportion of caregivers with primary studies, compared to those from Portugal. Spanish children were slightly younger than the rest. The Portuguese sample reported having a larger home (based on square meters) than the Spanish one. A higher proportion of Italian participants than the rest had an outdoor exit at home (e.g., garden or terrace). Compared to the Portuguese sample, a higher proportion of Spanish participants had an outdoor exit at home. Parental stress was significantly higher in the Italians compared to the Portuguese.

**Table 1:** Sample characteristics and equivalence by country

	Total (*n* = 624)	Italy (1) (*n* = 324)	Spain (2) (*n* = 159)	Portugal (3) (*n* = 141)	Test^a^	Effect size^b^	Post-hoc
*Parents*							
Female, *N* (%)	560 (89.7)	302 (93.2)	134 (84.3)	124 (87.9)	0.88**	.12	1 > 2
Age, *M* (*SD*)	42.20 (5.48)	42.40 (5.81)	42.11 (5.30)	41.82 (4.87)	1.21	–	–
Marital status, *N* (%)					8.49	–	–
Married	547 (87.7)	286 (88.3)	142 (89.3)	119 (84.4)			
Single	75 (12)	38 (11.7)	15 (9.4)	22 (15.6)			
Other	3 (0.3)	0 (0)	2 (1.3)	0 (0)			
Educational level, *N* (%)					56.32***	.21	
Ph.D./Master’s	171 (27.4)	65 (20.1)	44 (27.7)	62 (44)			3 > 1
Undergraduate	270 (43.3)	135 (41.7)	70 (44)	65 (46.1)			
Secondary school	158 (25.3)	111 (34.3)	33 (20.8)	14 (9.9)			1 > 3
Primary school	25 (4)	13 (4)	12 (7.5)	0 (0)			2 > 3
Monthly family income (euros)					4.25	–	–
Up to 999	29 (5.2)	14 (4.9)	8 (5.6)	7 (5.5)			
1000–1999	150 (26.9)	71 (24.9)	40 (27.8)	39 (30.5)			
2000–2999	194 (34.8)	105 (36.8)	43 (29.9)	46 (35.9)			
3000–4999	151 (27.1)	77 (27)	44 (30.6)	30 (23.4)			
5000 or more	33 (5.9)	18 (6.3)	9 (6.3)	6 (4.7)			
*Children*							
Female, *N* (%)	294 (47.1)	166 (51.2)	71 (44.7)	57 (40.4)	5.12	–	–
Age, *M* (*SD*)	8.94 (4.19)	9.18 (4.25)	8.12 (3.67)	9.31 (4.49)	7.08*	.01	1 > 2 1 > 3
*Living conditions*							
Square meters home, *M* (*SD*)	125 (59.18)	123.98 (58.98)	122.07 (56.08)	134 (64.24)	7.60*	.007	3 > 2
People living at home during quarantine,* M* (*SD*)	3.92 (.90)	3.98 (.93)	3.87 (.83)	3.84 (.89)	1.93	–	–
Outdoor exit (yes/no), *N* (%)	356 (57.1)	236 (72.6)	71 (44.1)	49 (34.8)	71.54***	.34	1 > 2 1 > 3 2 > 3
Parental stress, *M* (*SD*)	3.14 (.03)	3.23 (.97)	3.10 (.96)	2.99 (.98)	7.20*	.01	1 > 3

*M* Mean, *SD* Standard Deviation

ªCross-table (*χ*^2^) for categorical variables, and Kruskal–Wallis (*χ*^2^) for continuous variables

^b^Effect size = Cramer’s *V* for multi-categorical variables, and Epsilon-squared for continuous variables

***p < .001, **p < .01, *p < .05

### Procedure

The study was approved by the Ethics Board of the authors’ institution. Participants were recruited via social networks, including social media platforms (Facebook, LinkedIn, and Instagram) and researchers’ acquaintances (email), using a snowball sampling strategy. An online survey was created ad-hoc and distributed in each country (via Qualtrics or Google Forms). After the first evaluation, families were contacted by email. Because face-to-face evaluation was not allowed due to the COVID-19 health crisis, data were obtained using an online survey designed ad hoc for the current study. Before completing the survey, participants were provided with information about the objectives of the study, and informed consent was requested to access the assessment. Data were collected in all three countries using a snowball sampling strategy at three time-points: two weeks (Time 1), five weeks (Time 2), and eight weeks after the lockdown (Time 3). Italy and Spain followed mandatory confinement at all the time-points. At Time 3, Italy began a less restricted phase, opening parks and allowing visits to relatives. In Spain, Time 3 coincided with permission for children to go outside one hour a day, but gardens and public spaces were still closed. During the entire study, Portugal followed a general duty of home confinement. Schools were closed in all threes countries at the three assessments.

### Mesasures

#### Child Psychological Symptoms

The Impact Scale of the COVID-19 and Home Confinement on Children and Adolescents [11] was completed by parents. The scale included 31 items rated from 1 (*much less compared to before home confinement*) to 5 (*much more compared to before home confinement*). The global question was: “During the past few days, compared to before home confinement, have you noticed that your child…”. The scale included 10 items related to “anxiety” (e.g., “is worried”, “is afraid of COVID-19 infection”), 6 items related to “mood” (e.g., “is sad”), 5 items related to “sleep” (e.g., “is afraid to sleep alone”), 6 items related to “behavioral disturbances” (e.g., “argues with the rest of the family”), 2 items related to “Feeding” (e.g., “eats a lot”), and 2 items related to “cognitive disturbances” (e.g., “has difficulty concentrating”). Information provided in each item was coded into two categories (the child had worsened or had not worsened since before confinement). Cronbach’s alpha in this sample was high (α = 0.91): Anxiety (α = 0.89), Mood (α = 0.84), Sleep (α = 0.90), Behavioral disturbances (α = 0.85), Feeding (α = 0.27, which includes only two items that evaluate opposite aspects: No appetite and Eats a lot), and Cognitive disturbances (α = 0.75).

#### General Questionnaire

Participants also reported the caregiver’s age and gender, marital status, educational level, family income, and the children’s age and gender. Housing conditions were also evaluated (e.g., square meters and outdoor exits), and specific information was requested about the period of confinement (e.g., number of people living at home during confinement). Regarding the outdoor exist, having a garden or terrace was recoded as 1 (outdoor exit). Options “only windows,” “balcony,” and “other exits” were coded as 0 (no outdoor exit). Parental stress was also evaluated using a five-point scale: “How stressed do you perceive yourself?” (from 1 “Not at all” to 5 “A lot”).

### Data Analyses

The collected data were analyzed with SPSS for Windows-version 26. Nonparametric tests were used because the variables did not have a normal distribution, based on the Kolmogorov–Smirnov test (*p*-value < 0.05). The equivalence of sociodemographic variables among the samples recruited in the three countries was tested using chi-squared in cross-table (categorical variables) and Kruskal–Wallis test (continuous variables). The effect size of statistically significant comparisons was tested using Cramer’s *V* (multi-categorical variables) and Epsilon-squared (continuous variables). Cramer’s *V* was interpreted as follows: > 0.25 very strong, > 0.15 strong, > 0.10 moderate, > 0.05 weak, and > 0 none or very weak [13]. Epsilon-squared (ε^2^) was interpreted as follows: small effect sizes ranged from 0.01 to < 0.08, medium effect sizes ranged from 0.08 to < 0.26, and large effect sizes ranged from ≥ 0.26.

Spearman correlations were calculated to analyze the relationship between continuous variables. Multivariate analyses were also performed to determine the independent factors associated with anxiety, mood problems, and sleep, behavioral, eating, and cognitive disturbances (dependent variables). Factors significantly related to dependent variables were entered in a generalized multivariate linear model, including time, country, child’s sex and age, parental stress, and housing conditions (square meters and outdoors exit). Estimated marginal means of psychological reactions (anxiety, mood, sleep, behavioral disturbances, eating disturbances, and cognitive disturbances), considering the independent categorical variables (time, country, child’s sex, and outdoor exit) were calculated. All statistical analyses were estimated with the GENLIN syntax. Measurements of all the participants were included, even if they had not responded to all three assessments. Differences were considered statistically significant at a *p*-value of < 0.05.

## Results

### Attrition

**Fig. 1:**
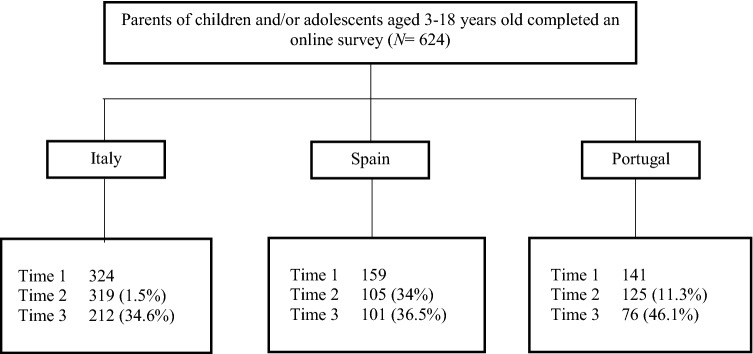
Flowchart of participants by country

Figure 1 illustrates the flow of participants during the three time-points by country. Dropout rate at Time 2 was 11.9% (*n* = 74). The odds of dropping out were higher in Spain, compared to Italy (χ^2^ = 109, *p* ≤ 0.001, Cramer’s *V* = 0.41). Dropout rate at Time 3 was 37.7% (*n* = 235). The odds of dropping out were similar across countries (χ^2^ = 5.69, *p* = 0.06). Dropout rates at Time 2 and Time 3 were unrelated to caregivers’ sex and age, educational level, marital status, monthly family income, and symptoms of anxiety, mood, sleep, behavioral, eating, and cognitive disturbances.

### Psychological Effects and Related Factors

Spearman correlations between psychological reactions and child’s age, square meters home, number of people living at home during confinement, and parental stress were calculated (Table 2). Because the number of people living at home during quarantine did not reach a significant correlation with psychological or behavioral symptoms (*p* > 0.05), it was not included in the models. Younger children were more likely to present sleep and behavioral disturbances than older ones. Children who lived in larger homes tended to present more symptoms of mood and behavioral disturbances but these correlations were very low. Parental stress was directly and statistically related to more children’s psychological symptoms.

**Table 2:** Correlations among continuous variables with confidence intervals

Variable	1	2	3	4	5	6
Child’s age	− .01	.02	− .20**	− .09*	− .03	.02
	[− .09, .07]	[− .06, .10]	[− .27, − .12]	[− .17, − .01]	[− .10, .05]	[− .06, .10]
Square meters home	.07	.09*	.00	.08*	.03	.08*
	[− .00, .15]	[.01, .17]	[− .08, .08]	[.00, .16]	[− .05, .10]	[.00, .16]
Parental stress	.32**	.27**	.12**	.25**	.14**	.21**
	[.25, .39]	[.20, .34]	[.04, .20]	[.17, .32]	[.06, .22]	[.13, .28]

Values in brackets indicate the 95% confidence interval for each correlation. The confidence interval is a plausible range of population correlations that could have caused the sample correlation

1 = Anxiety, 2 = Mood, 3 = Sleep disturbances, 4 = Behavioral disturbances, 5 = Eating disturbances, 6 = Cognitive disturbances

*p < .05; **p < .01

### Evolution of Psychological Effects Across Time on Children from Italy, Spain, and Portugal

#### Italy

The level of anxiety (*OR* = 3.79; 95% CI [2.90, 4.95], *p* ≤ 0.001) and mood symptoms (*OR* = 1.98; 95% CI [1.63, 2.39], *p* ≤ 0.001), sleep (*OR* = 1.48; 95% CI [1.30, 1.70], *p* ≤ 0.001), behavioral (*OR* = 1.44; 95% CI [1.21, 1.72], *p* ≤ 0.001), and cognitive disturbances (*OR* = 1.18; 95% CI [1.09, 1.28], *p* ≤ 0.001) increased at Time 2 compared to Time 1. At Time 3, anxiety (*OR* = 1.43; 95% CI [1.04, 1.95], *p* = 0.02) and mood symptoms (*OR* = 0.52; 95% CI [0.41, 0.67], *p* ≤ 0.001) decreased compared to Time 2 but eating disturbances increased during this period (*OR* = 0.88; 95% CI [0.80, 0.96], *p* = 0.005). When comparing Time 1 and Time 3, an increase of anxiety (*OR* = 2.65; 95% CI [1.90, 3.69], *p* ≤ 0.001) and mood symptoms (*OR* = 1.88; 95% CI [1.47, 2.41], *p* ≤ 0.001), sleep (*OR* = 1.65; 95% CI [1.37, 1.99], *p* ≤ 0.001), behavioral (*OR* = 1.55; 95% CI [1.24, 1.93], *p* ≤ 0.001), eating (*OR* = 1.16; 95% CI [1.06, 1.26], *p* = 0.001), and cognitive disturbances (*OR* = 1.28; 95% CI [1.15, 1.42], *p* ≤ 0.001) were observed over time.

#### Spain

The level of anxiety marginally increased at Time 2 compared to Time 1 (*OR* = 3.88; 95% CI [0.85, 17.63], *p* = 0.07). No significant changes were observed in mood symptoms, sleep, behavioral, eating, and cognitive disturbances from Time 1 to Time 2. When comparing Time 2 and Time 3, anxiety symptoms (*OR* = 2.48; 95% CI [1.28, 4.78], *p* = 0.007) decreased but no significant changes were observed in other symptoms. A decrease of anxiety symptoms was also observed from Time 1 to Time 3 (*OR* = 0.40; 95% CI [0.20, 0.77], *p* = 0.007).

#### Portugal

No significant changes were observed over time in anxiety and mood symptoms, sleep, behavioral, eating, and cognitive disturbances. Only a marginally significant decrease of eating disturbances was observed from Time 1 to Time 3 (*OR* = 0.88; 95% CI [0.77, 1], *p* = 0.06).

### Differences by Country Across Time

Table 3 presents estimated marginal means of psychological reactions (anxiety, mood problems, sleep problems, behavioral disturbances, eating disturbances, and cognitive disturbances) by time, country, child’s sex, and outdoor exit (yes/no). Except for the level of eating disturbances, all the psychological symptoms differed across time and some differences by country were also observed.

**Table 3:** Estimated marginal means of psychological reactions for categorical independent variables (country, child’s sex and age, and outdoor exit) by time

	Anxiety	Mood	Sleep	Behavioral disturbances	Eating disturbances	Cognitive disturbances
	Mean (95% CI) Range (0–10)	Mean (95% CI) Range (0–6)	Mean (95% CI) Range (0–5)	Mean (95% CI) Range (0–6)	Mean (95% CI) Range (0–2)	Mean (95% CI) Range (0–2)
Time [1]	2.75 (2.59, 2.91)	1.88 (1.78, 1.99)	.69 (.63, .75)	1.63 (1.53, 1.73)	.32 (.30, .34)	.40 (.37, .42)
Time [2]	4.08 (3.79, 4.36)	2.55 (2.35, 2.75)	1.09 (.94, 1.23)	2 (1.81, 2.19)	.35 (.28, .41)	.56 (.48, .64)
Time [3]	2.77 (2.27, 3.28)	2.27 (1.27, 3.27)	.79 (.04, 1.58)	.96 (− .73, 2.65)	.12 (− .21, .47)	.73 (.33, 1.14)
Country [Portugal]						
Time [1]	2.07 (1.74, 2.39)	2.05 (1.83, 2.26)	.65 (.55, .76)	1.69 (1.51, 1.88)	.37 (.33, .40)	.40 (.36, .43)
Time [2]	2.50 (2.10, 2.91)	2.20 (1.92, 2.47)	.81 (.63, 1)	1.74 (1.50, 1.99)	.39 (.30, .48)	.48 (.38, .57)
Time [3]	2.36 (1.82, 2.90)	2.04 (1.69, 2.39)	.56 (.33, .78)	1.68 (1.39, 1.97)	.26 (.15, .37)	.41 (.28, .55)
Country [Italy]						
Time [1]	1.68 (1.51, 1.85)	2.05 (1.83, 2.26)	.68 (.60, .76)	1.45 (1.35, 1.55)	.32 (.31, .33)	.40 (.37, .42)
Time [2]	3.33 (3.07, 3.60)	2.64 (2.46, 2.82)	1.02 (.88, 1.16)	1.75 (1.59, 1.91)	.29 (.23, .34)	.53 (.47, .60)
Time [3]	3 (2.69, 3.30)	2.56 (2.34, 2.77)	1.08 (.90, 1.25)	1.75 (1.56, 1.94)	.40 (.33, .47)	.58 (.49, .67)
Country [Spain]						
Time [1]	2.20 (1.94, 2.46)	1.66 (1.48, 1.84)	.82 (.69, .95)	1.78 (1.58, 1.97)	.31 (.33, .40)	.42 (.38, .46)
Time [2]	3.15 (2.71, 3.58)	2.10, 1.80, 2.41)	1.10 (.87, 1.34)	1.99 (1.71, 2.26)	.30 (.20, .39)	.60 (.49, .72)
Time [3]	1.95 (1.34, 2.56)	1.99 (.89, 3.08)	.89 (.38, 1.40)	1.18 (− .40, 2.78)	.31 (− .05, .69)	.62 (.18, 1.05)
Child’s sex						
Time [1]						
Girls	2.66 (2.42, 2.90)	1.88 (1.73, 2.03)	.70 (.60, .79)	1.54 (1.41, 1.67)	.32 (.29, .35)	.38 (.34, .42)
Boys	2.83 (2.63, 3.03)	1.88 (1.75, 2.01)	.68 (.60, .76)	1.71 (1.59, 1.84)	.32 (.30, .35)	.42 (.38, .45)
Time [2]						
Girls	3.99 (3.66, 4.33)	2.55 (2.33, 2.77)	1.09 (.94, 1.25)	1.91 (1.70, 2.13)	.34 (.28, .41)	.54 (.45, .63)
Boys	4.16 (3.85, 4.46)	2.55 (2.33, 2.77)	1.08 (.92, 1.24)	2.08 (1.88, 2.29)	.35 (.28, .42)	.58 (.50, .66)
Time [3]						
Girls	2.70 (2.13, 3.28)	2.29 (1.31, 3.27)	.81 (.02, 1.59)	.88 (− .81, 2.57)	.12 (− .21, .46)	.71 (.30, 1.12)
Boys	2.87 (2.39, 3.35)	2.28 (1.31, 3.26)	.79 (.01, 1.57)	1.05 (− .63, 2.73)	.13 (− .20, .46)	.75 (.35, 1.16)
Outdoor exit						
Time [1]						
Yes	2.73 (2.50, 2.96)	1.87 (1.71, 2.02)	.68 (.58, .77)	1.57 (1.43, 1.70)	.31 (.28, .34)	.39 (.36, .43)
No	2.76 (2.53, 2.99)	1.90 (1.75, 2.05)	.70 (.61, .80)	1.68 (1.55, 1.82)	.33 (.30, .36)	.40 (.36, .44)
Time [2]						
Yes	4.06 (3.72, 4.40)	2.54 (2.30, 2.77)	1.07 (.92, 1.23)	1.94 (1.72, 2.16)	.34 (.27, .41)	.56 (.47, .64)
No	4.09 (3.77, 4.40)	2.56 (2.34, 2.78)	1.10 (.94, 1.27)	2.06 (1.85, 2.26)	.35 (.29, .42)	.56 (.47, .65)
Time [3]						
Yes	2.77 (2.25, 3.29)	2.27 (1.29, 3.25)	.78 (.004, 1.454)	.90 (− .78, 2.60)	.11 (− .21, .45)	.73 (.32, 1.13)
No	2.80 (2.25, 3.34)	2.30 (1.32, 3.27)	.81 (.03, 1.59)	1.02 (− .66, 2.71)	.13 (− .20, .47)	.73 (.33, 1.14)

Anxiety significantly increased from Time 1 to Time 2 (*OR* = 3.78; 95% CI [2.90, 4.91], *p* ≤ 0.001), and decreased from Time 2 to Time 3 (*OR* = 3.69; 95% CI [2.09, 6.50], *p* = 0.003). Differences by country across time were observed. At Time 1, Spanish children presented more anxiety compared to the Italian ones (*OR* = 1.68; 95% CI [1.20, 2.34], *p* = 0.002). At Time 2, the level of anxiety in children from Italy (*OR* = 0.43; 95% CI [0.26, 0.70], *p* = 0.001) and Spain (*OR* = 1.89; 95% CI [1.06, 3.40], *p* = 0.03) was higher compared to the Portuguese ones. At Time 3, Italian children reached a higher level of anxiety compared to the Spanish (*OR* = 0.35; 95% CI [0.17, 0.69], *p* = 0.003) and the Portuguese ones (*OR* = 0.52; 95% CI [0.28, 0.98], *p* = 0.04).

Mood symptoms significantly increased from Time 1 to Time 2 (*OR* = 1.95; 95% CI [1.61, 2.35], *p* = 0.005). At Time 1, Italian (*OR* = 0.74; 95% CI [0.59, 0.92], *p* = 0.007) and Portuguese children (*OR* = 0.67; 95% CI [0.51, 0.89], *p* = 0.007) presented more mood symptoms compared to the Spanish ones. At Time 2, the level of mood symptoms was higher in Italian children than in Spanish (*OR* = 0.58; 95% CI [0.41, 0.83], *p* = 0.003) and Portuguese ones (*OR* = 0.64; 95% CI [0.46, 0.89], *p* = 0.008). At Time 3, Italian children showed more mood symptoms compared to the children from Portugal (*OR* = 0.59; 95% CI [0.39, 0.90], *p* = 0.01).

Sleep disturbances significantly increased from Time 1 to Time 2 (*OR* = 1.49; 95% CI [1.30, 1.70], *p* ≤ 0.001) but differences by country were only observed at Time 3, with more sleep problems in Italian children than in the Portuguese ones (*OR* = 0.59; 95% CI [0.44, 0.79], *p* ≤ 0.001).

Regarding behavioral disturbances, they significantly increased from Time 1 to Time 2 (*OR* = 1.45; 95% CI [1.21, 1.73], *p* ≤ 0.001). At Time 1, Portuguese (*OR* = 1.27; 95% CI [1.03, 1.57], *p* = 0.02) and Spanish children (*OR* = 1.38; 95% CI [1.11, 1.72], *p* = 0.003) showed a higher level of behavioral disturbances than Italian ones but no statistically significant differences by country were found at Times 2 and 3.

The level of eating disturbances did not differ across time, but differences by country across time were observed. At Time 1, Portuguese children reached a significantly higher level of eating disturbances compared to the Italian (*OR* = 1.04; 95% CI [1, 1.08], *p* = 0.02) and Spanish ones (*OR* = 0.94; 95% CI [0.90, 0.99], *p* = 0.02). Although at Time 2, Portuguese children presented more eating disturbances than the Italian ones (*OR* = 1.11; 95% CI [1, 1.23], *p* = 0.04), at Time 3, children from Italy presented a higher level compared to the Portuguese ones (*OR* = 0.87; 95% CI [0.76, 0.99], *p* = 0.03).

Cognitive disturbances significantly increased from Time 1 to Time 2 (*OR* = 1.17; 95% CI [1.08, 1.27], *p* ≤ 0.001), but no differences were observed across countries.

### Parental Stress, Children’s Age and Sex, and Housing Conditions

Parental stress was related to all children´s psychological symptoms, except for eating disturbances. Specifically, a higher level of parental stress was related to a higher level of anxiety symptoms (*OR* = 1.62; 95% CI [1.38, 1.89], *p* ≤ 0.001), observing a statistically significant Time x Parental Stress interaction at Time 1 (*OR* = 1.43; 95% CI [1.22, 1.68], *p* ≤ 0.001) and Time 2 (*OR* = 2.05; 95% CI [1.73, 2.42], *p* ≤ 0.001). Also, a positive relation between the level of parental stress and the level of mood symptoms was found (*OR* = 1.39; 95% CI [1.26, 1.52], *p* ≤ 0.001), with a significant interaction between Time x Parental Stress at Time 1 (*OR* = 1.34; 95% CI [1.21, 1.48], *p* ≤ 0.001) and Time 2 (*OR* = 1.50; 95% CI [1.25,1.80], *p* ≤ 0.001). Regarding sleep disturbances, parental stress was positively related to sleep problems (*OR* = 1.09; 95% CI [1.02, 1.17], *p* = 0.007), observing that the Time x Parental Stress interaction was only significant at Time 2 (*OR* = 1.18; 95% CI [1.09, 1.28], *p* ≤ 0.001). Behavioral disturbances were also positively related to the level of parental stress (*OR* = 1.35; 95% CI [1.24, 1.47], *p* ≤ 0.001), observing that the Time x Parental Stress interaction was significant at Time 1 (*OR* = 1.31; 95% CI [1.20, 1.44], *p* ≤ 0.001) and Time 2 (*OR* = 1.43; 95% CI [1.22,1.68], *p* ≤ 0.001). Moreover, a higher level of parental stress was related to a higher level of cognitive disturbances (*OR* = 1.07; 95% CI [1.04, 1.11], *p* ≤ 0.001), with a significant Time x Parental Stress interaction at Time 1 (*OR* = 1.07; 95% CI [1.03, 1.10], *p* ≤ 0.001) and Time 2 (*OR* = 1.11; 95% CI [1.04,1.19], *p* = 0.002).

Child’s age and sex were not related to the level of psychological symptoms, except for sleep and behavioral problems. Younger children presented more sleep disturbances (*OR* = 0.97; 95% CI [0.95, 0.98], *p* = 0.001) compared to the older ones, finding a significant Time x Child’s Age interaction at Time 1 (*OR* = 0.97; 95% CI [0.96, 0.99], *p* = 0.01). Also, boys presented more behavioral disturbances compared to girls (*OR* = 0.84; 95% CI [0.71, 0.99], *p* = 0.04), and younger children more than the older ones (*OR* = 0.97; 95% CI [0.95, 0.99], *p* = 0.01). The Time x Child’s Sex interaction was significant from Time 1 to Time 2 (*OR* = 1.74; 95% CI [1.34, 2.25], *p* ≤ 0.001), and the Time x Child’s Age interaction was significant at Time 3 (*OR* = 0.96; 95% CI [0.94, 0.98], *p* = 0.001).

Housing conditions were only related to the level of eating disturbances. Specifically, more square meters was related to higher level of eating disturbances (*OR* = 1; 95% CI [0.99, 1], *p* = 0.02), with a statistically significant Time x Square Meters interaction at Time 2 (*OR* = 0.99; 95% CI [0.99, 1], *p* = 0.03).

## Discussion

The present study aimed to examine the evolution of the psychological impact of the COVID-19 home confinement in children and adolescents from three European countries that followed different restrictions. Italy, Spain, and Portugal applied the lockdown beginning the second week of March 2020, but strategies to slow down the spread of the COVID-19 varied in each country, affecting the children´s lives differently.

The results of the study show that, in general, the psychological symptoms increased from the Time 1 (two weeks after the lockdown) to the Time 2 (three weeks later). Specifically, significant differences were found in all the symptoms explored, except for eating disturbances. A decrease in most symptoms was shown from Time 2 to the last one (eight weeks after starting the home confinement), but significant differences were only found in the level of anxiety.

When examining each country, a different pattern was found. Italy was the first European country affected by the pandemic and also the first to implement a lockdown. Examination of the evolution highlights an increase of the psychological symptoms as the confinement was prolonged. Specifically, anxiety, mood, sleep, behavioral, and cognitive disturbances were more likely to emerge after more weeks of confinement. Eating problems are also a symptom that appears the longer the confinement is. As previous studies have shown, long confinements are associated with more psychological impact [14], so it can be expected that a higher level of symptoms will show in the last assessment compared to the beginning of the confinement. Moreover, although the State of Alarm was declared in Italy initially from March 10th to April 3rd, it was extended several times past its deadline until October 15th. Previous studies have shown that an extension in quarantine increases the frustration and demoralization of confined people [15], so the various lockdown extensions may be influencing the mental health of Italian children. Also, despite that in the middle of the last assessment of the present study, a less restricted phase began in Italy, many activities were still forbidden due to the State of Alarm that was still maintained. Although parks were re-opened and visits to relatives were allowed, schools were still closed, so social contact with friends was not yet possible, providing the children with an environment still far from being considered normalized.

In the Spanish sample, no significant differences were found between the three measurements, except for anxiety, which was more likely five weeks after the confinement than at the first assessment, and decreased from the second to the third assessment. Anxiety symptoms are commonly reported manifestations related to children’s stress during the COVID-19 pandemic [5, 6, 10, 16]. In line with these findings, it is not surprising that, in the present study, the level of Spanish children with anxiety symptoms increased after five weeks of being confined. When observing the results, it seems that psychological symptoms increased from the first to the second assessment, and then there was a decrease at the last one. Although significant differences were only found in anxiety, this pattern should be taken into account to provide some explanation. Spain applied one of the most restricted confinements in Europe. When the first and second assessments were obtained, Spanish children were not allowed to go outside, and more children were affected by the restrictions imposed. After six weeks of confinement, and coinciding with the third assessment, children had permission to go outside but with specific rules: only one hour a day, once a day, no more than 1 km from home, and accompanied by only one adult. Outdoor play has been associated with many benefits, highlighting its importance in children’s healthy development [17]. The opportunity to go outdoors could improve the Spanish children’s mental health, although restrictions were still too harsh to make a difference in most symptoms. Despite that more research is needed, it seems that the improvement in the emotional and behavioral status of the Spanish children could be explained by the change in the restrictions, which allowed children to go outside, enjoying being outdoors again after six weeks of confinement.

Portuguese children showed a different pattern from the Italian and Spanish children. No significant differences were found, although a slight increase in psychological symptoms was observed after five weeks of confinement, and a slight decrease was perceived at the last assessment. Despite its closeness to Spain, Portugal managed to effectively apply measures to slow down COVID-19, promoting social distance and following a general duty of home confinement. As the results show, the impact of the COVID-19 situation on Portuguese children was lower, revealing nonsignificant differences over time. When the third assessment was applied, the emergency state had been canceled in Portugal, and less restricted measures were applied (despite that most of the children remained at home and did not go to school), providing the children with a more “normalized” environment that helped to preserve their mental health.

Comparison across countries showed interesting results. The results of the interaction confirmed the pattern found for each country: Italian children were more psychologically impacted by home confinement over time, Portuguese children were in general the best adapted to the situation, and Spanish children were more psychologically impacted during the first five weeks of confinement but their symptoms decreased at the last assessment. However, although Italian children were more affected over time compared to the other countries, it seems that they were better adapted in the first weeks after the lockdown. In fact, at the first time the level of psychological symptoms was lower in Italians than in children from the other countries, except for mood symptoms, but it increased from the first to the last assessment. This finding may imply that in general Italian children were better adapted than Spanish and Portuguese children in the first weeks of confinement, but their wellbeing worsened as of week five. The long confinement and the severe surrounding environment maintained over time, with a high number of infections and deaths, may explain why the psychological wellbeing of the Italian children got worse as the confinement was prolonged, although their adjustment to the situation was better than that of the other two countries in the first weeks after the lockdown. Contrarily, Portuguese children were the best adapted eight weeks after the confinement, showing less anxiety, and fewer mood, sleep, and eating disturbances than Italian ones. Although the longer the confinement, the more impact it may have on mental health, less restricted measures could explain the differences found between these two countries.

Parental stress was related to higher levels of all psychological symptoms in children, except for eating disturbances. Previous studies have shown the relation between parent´s stress and children’s psychological problems during the pandemic (e.g., [3]). Facing a home confinement implies a high level of parental stress, which influences parenting responses and parents’ ability to be supportive [3, 18], negatively affecting their children’s well-being (e.g., [19, 20]). In our study, parental stress was associated with a high level of symptoms in children only during the two first assessments. Although more research is needed, it seems that parents managed to face the stressors more effectively as the pandemic continues, as parental stress is not related to their children’s well-being eight weeks after the confinement. A relationship between children’s characteristics and their psychological symptoms was also found. Specifically, sleep and behavioral problems were more probable in younger children than in older ones, and boys were more likely to present behavioral problems than girls. These findings are in line with previous studies concluding that externalized problems are more likely in males than in females (e.g., [21]), and that sleep (e.g.,[22]) and externalizing behavior (e.g., [23]) decrease with age.

This study has as its main limitation the sample size, which may limit the interpreting of some results. Also, it should be taken into account that the Feeding subscale had a very low reliability. Although it would be preferable to use information provided by the children, it was not possible to apply a face-to-face assessment. As behavioral problems were being assessed, obtaining the information from the children was not recommended, so we decided that the parents were the best informants in this situation. Despite the desirability of a multi-informant procedure, parents’ report of their children’s symptoms is considered valid [24] and has been a widely used procedure to request information in investigations during confinement (e.g., [3]). A main finding provided in this study is that home confinement affected each country differently over time, so a common pattern was not found. Differences in restrictions imposed, and the health emergency in each country (number of infections or deaths) could explain the impact on the children’s psychological wellbeing. Italy was the best-adapted country in the first weeks after the lockdown, but the most psychologically impacted by confinement in the last weeks of confinement, with an increase of the symptoms over time. Spain, which reached a higher level of symptoms in the first weeks compared to Italy, showed differences in anxiety as the confinement was prolonged, with a decrease at the last assessment. Contrary to Italy, no differences were found in Portugal across time, thus indicating their good adjustment to the situation.

Some practical implications are derived from the findings. First, as long confinements affect children [14], restricting quarantine duration is recommended. Although it is necessary to deal with the infections, home confinement impacts children’s and adolescents’ mental health negatively. So, when a quarantine must be implemented, children should be provided with psychological skills to help them to cope with such a stressful situation. Second, following the example of Portugal, less restricted measures should be applied during confinements to prevent psychological problems in children.

## Summary

The coronavirus disease emerged in the city of Wuhan (China) in December 2019, but it spread rapidly. A lockdown was declared in the first term of 2020 in many European countries aiming to slow down the spread of the COVID-19. The psychological effects of home confinement in European children have been described, finding emotional and behavioral problems during the COVID-19 confinement, but there is a lack of longitudinal research examining the impact of the confinement over time. The present study analyzes the evolution of the psychological wellbeing of Italian, Spanish, and Portuguese children and adolescents aged 3 to 18 years old. Parents completed the “Impact Scale of COVID-19 and Home Confinement on Children and Adolescents” two, five, and eight weeks after the lockdown, finding a different pattern for each country. Children from Italy, the first European country that applied a lockdown, were better adapted than Spanish and Portuguese children the first two weeks after confinement, but they were more psychologically impacted by home confinement in the eight-week assessment. Portuguese children, who followed a general duty of home confinement, were the best adapted to the situation, with no significant differences across time. A significant change was found in anxiety symptoms in Spanish children, with a decrease at the last assessment. Findings suggest that long confinements and hard restrictions affect children, so following the example of Portugal, less restricted measures should be applied during confinements. Also, prevention measures should be applied during confinements to prevent psychological problems in children.
